# The Tariff on Dyestuffs, and Drug Prices

**Published:** 1917-02

**Authors:** 


### THE TARIFF ON DYESTUFFS, AND DRUG PRICES.

Owing to the interdependence of dyestuffs and medicinal chemicals derived from coal-tar (the so-called coal-tar synthetics), any conditions influencing the production of the former necessarily are of interest to physicians. Most coal-tar "synthetics" have been developed either in the attempt to create new markets for the intermediate products in dye manufacture, or to utilize the by-products from the color industry. Dyes are extensively used in the cotton, leather, paint, silk, wall-paper and woolen industries, and at the beginning of the present European war, American manufactures were producing less than fifteen per cent. of the quantities used in this country. Of the foreign makers, Germany produced by far the largest amount. In that country the industry had grown to vast proportions, partly as a result of the efforts of manufacturers to utilize the results of chemical research, and especially because of the paternal attitude of the German government toward home industries and the development of trade. After the war broke out, the prices of medicinal chemicals advanced enormously, as may be seen by a few comparisons, the prices being for pounds.

ADVANCE IN PRICES OF MEDICINAL CHEMICALS.

| Drugs. | Price per Pound. August, 1914 | 1915 | 1916 |
|---|---|---|---|
| Phenol (carbolic acid) | $0.15 | $1.50 | $0.75 |
| Salicylic acid | 0.25 | 2.00 | 3.00 |
| Aspirin (acetylsalicylic acid) | 6.50 | 8.80 | 13.00 |
| Sodium salicylate | 0.39 | 2.00 | 3.00 |
| Salol | 0.75 | 2.60 | 6.50 |
| Acetphenetidin | 0.80 | 6.50 | 30.00 |
| Resorcin | 1.05 | 2.60 | 24.00 |
| Antipyrin | 3.75 | 15.00 | 32.00 |
| Phenolphthalein | 1.85 | 5.25 | 15.50 |
| Methylene blue | 1.40 | 3.25 | 26.00 |

It is presumable that these advances in prices have not been made necessary in all cases but that values have been forced up by manufacturers and speculators. For example, phenol (the basis of salicylic acid) is now manufactured here and its price is but half of that of a year ago, yet the prices of salicylic acid and aspirin have advanced. Inasmuch as aspirin is doubtless now made in the United States, presumably from salicylic acid which, in turn, had American-made phenol as a basis, the prices of phenol and aspirin should be parallel.

The revenue bill, which has just been passed by Congress, contains provisions designed to foster and maintain a dyestuff industry in this county. The bill admits parent dye materials and parent coal-tar medicinals free, but assesses fifteen per cent. ad valorem and two and five tenths cents a pound specific on intermediates, and thirty per cent. ad valorem and five cents a pound specific on finished products. The new tariffs become effective at once, but there are provisions for a graded reduction in rates under certain specified conditions. Experience with previous tariffs has shown that, if intermediates are given preferred rates, the goods will be imported so nearly finished that they need but a mere touch for completion, so that the American treasury and American industry both suffer.

The revenue bill also contains an "antidumping" clause. This forbids the sale in this country of imported goods at prices less than those for which they are sold in the country of issue. For years a provision of this kind has been sought in connection with patent legislation but, as passed in the revenue bill, it has broader applications than if applicable only to patented goods.

At present benzene (benzol, $C_6H_6$), phenol and toluene (the raw products of many dyes) are being manufactured in the United States in large quantities, but owing to the demand for them in the explosive industries, the prices are abnormally high. As soon as the war closes, these products probably will be largely diverted into dyestuffs channels, and prices may be expected to fall. The effect of the tariffs, however, on the cost of medicinal chemicals after the war (discounting speculative features) will be a tendency toward maintaining prices at higher levels than before the war. Under the stimulation of the present high prices and the guarantees of protection after the war, it is to be hoped that American manufacturers will soon produce all of the dyes and coal-tar synthetics needed for home consumption. —*Journal A. M. A.*, September, 1916.

